# Supplementary figures and images for: Saikosaponin A alleviates depressive-like behavior induced by reserpine in mice by regulating gut microflora and inflammatory responses
Source: PLoS One. 2025 Feb 10;20(2):e0311207. doi: 10.1371/journal.pone.0311207 (PMC11809902; doi:10.1371/journal.pone.0311207)

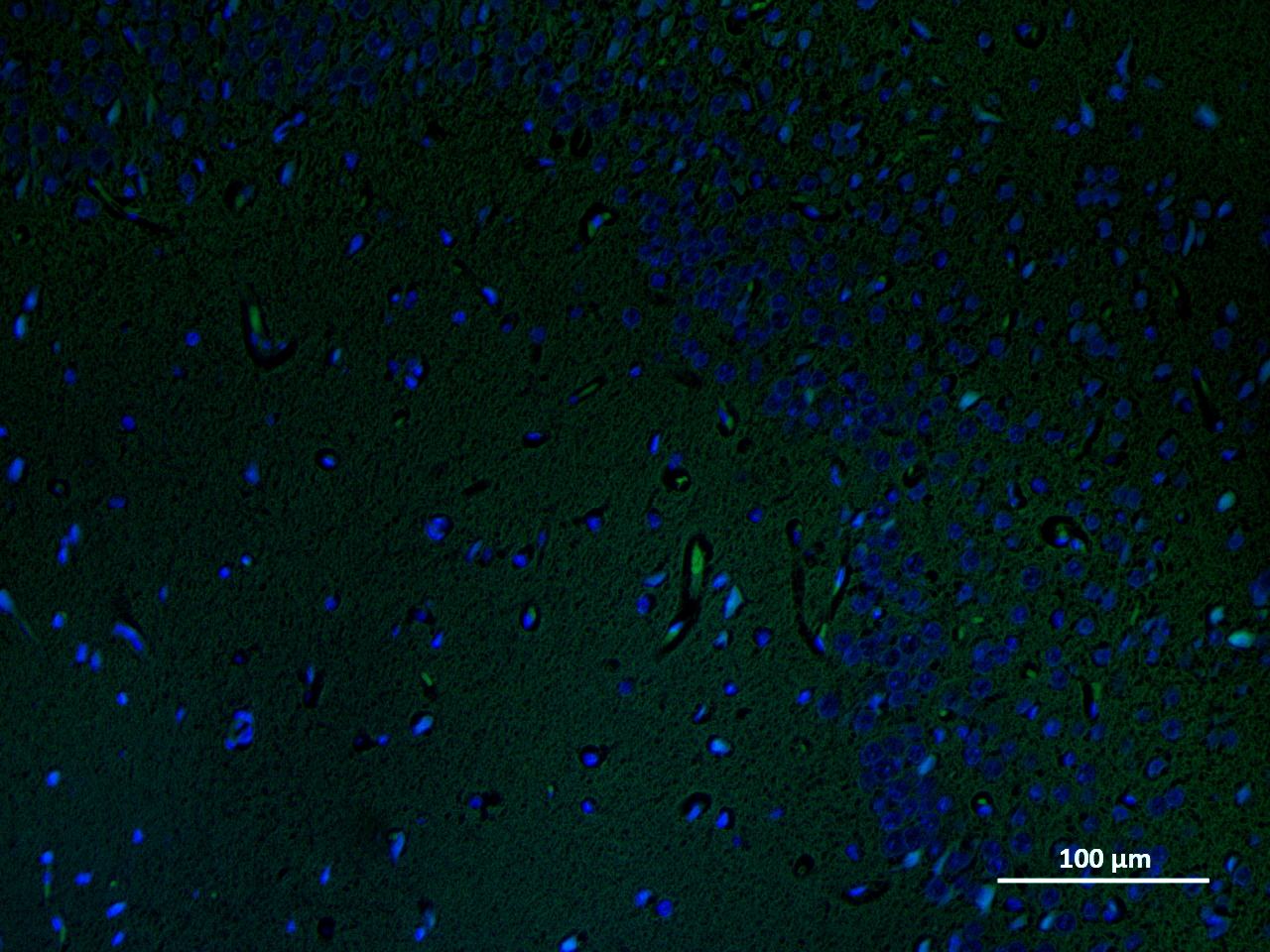

Supplement: S1 Data — (ZIP) [file pone.0311207.s001.zip › CG FJB-1 200.jpg]

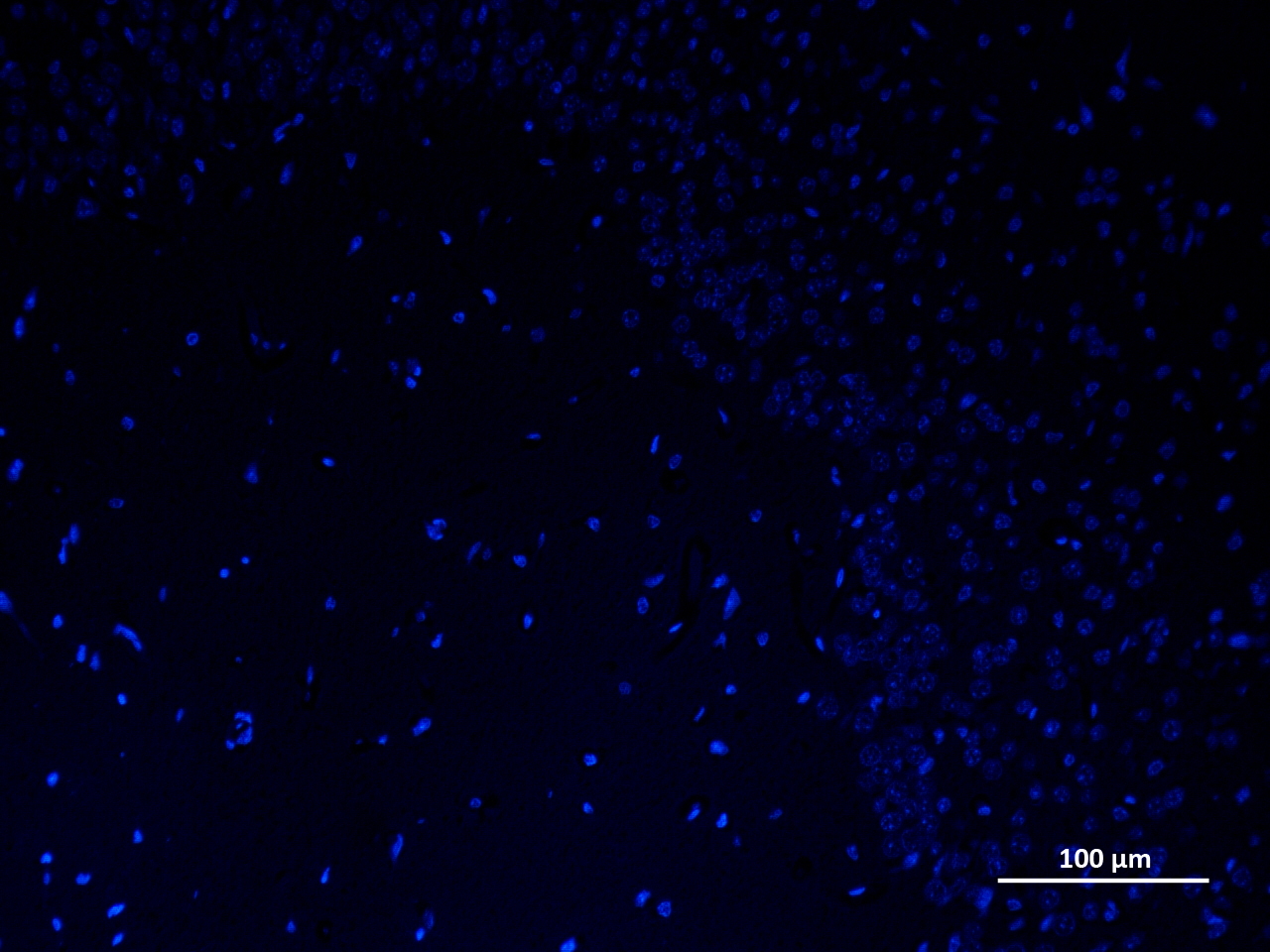

Supplement: S1 Data — (ZIP) [file pone.0311207.s001.zip › CG FJB-1-1 200.jpg]

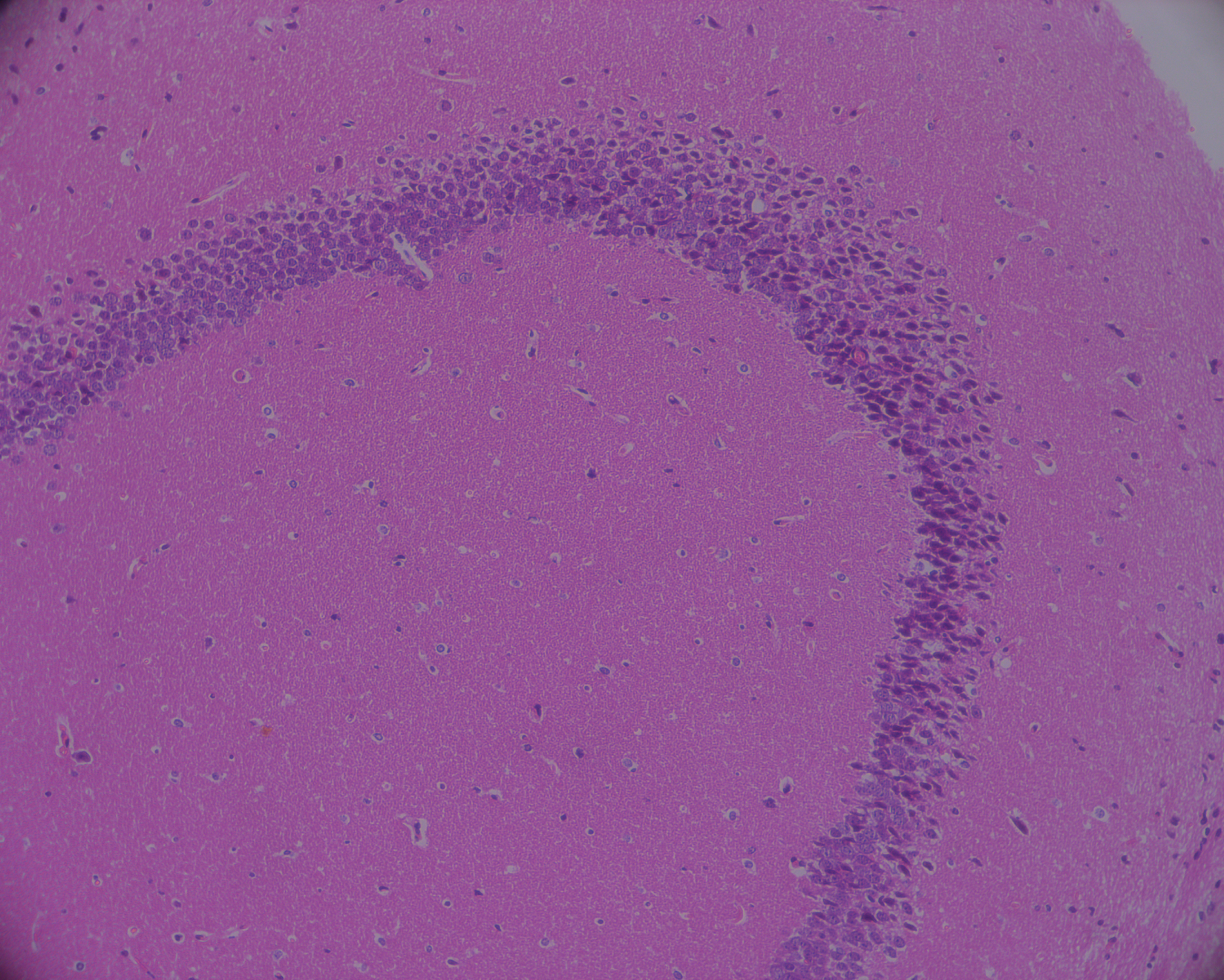

Supplement: S1 Data — (ZIP) [file pone.0311207.s001.zip › HE CG.jpg]

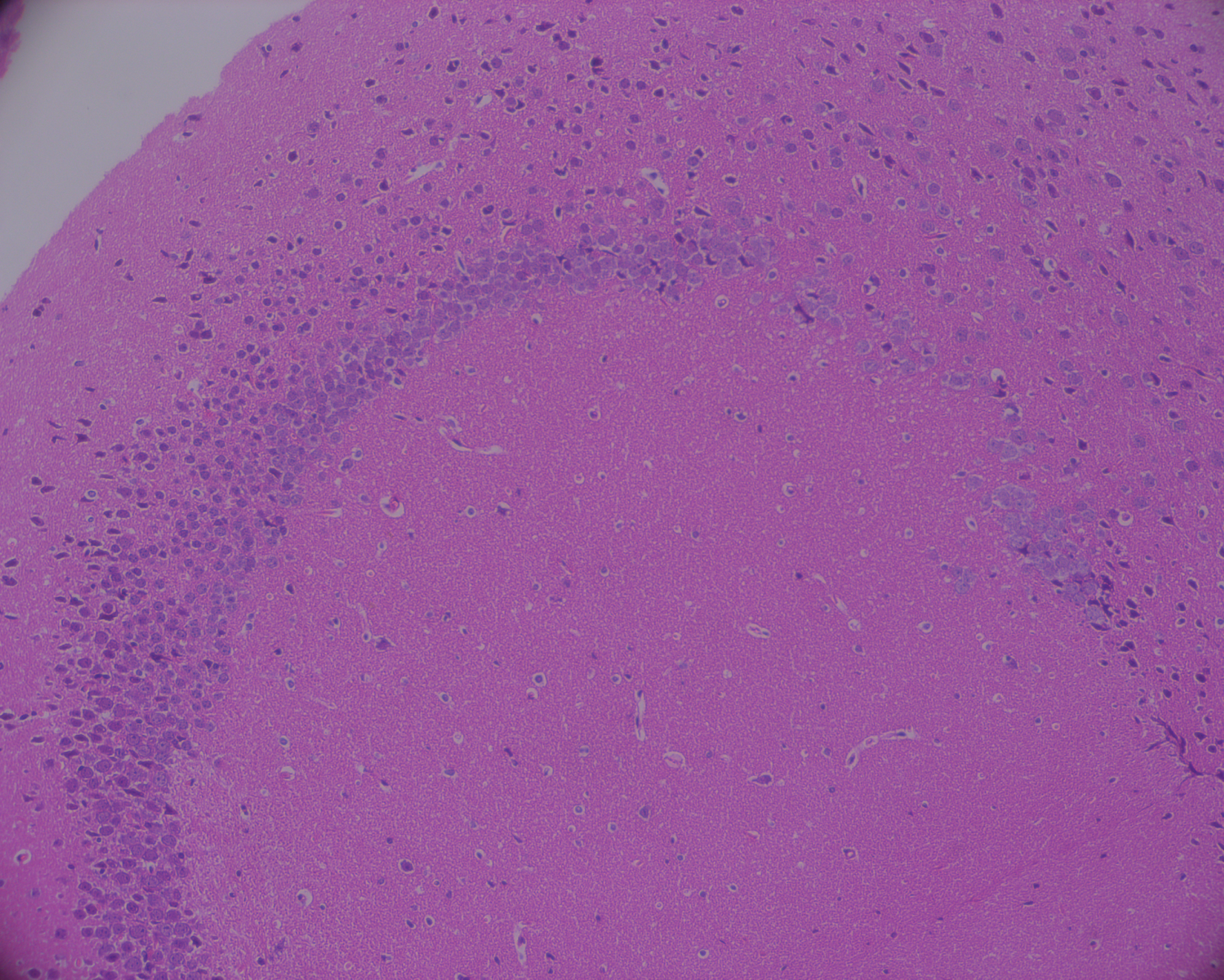

Supplement: S1 Data — (ZIP) [file pone.0311207.s001.zip › HE RSP.tif]

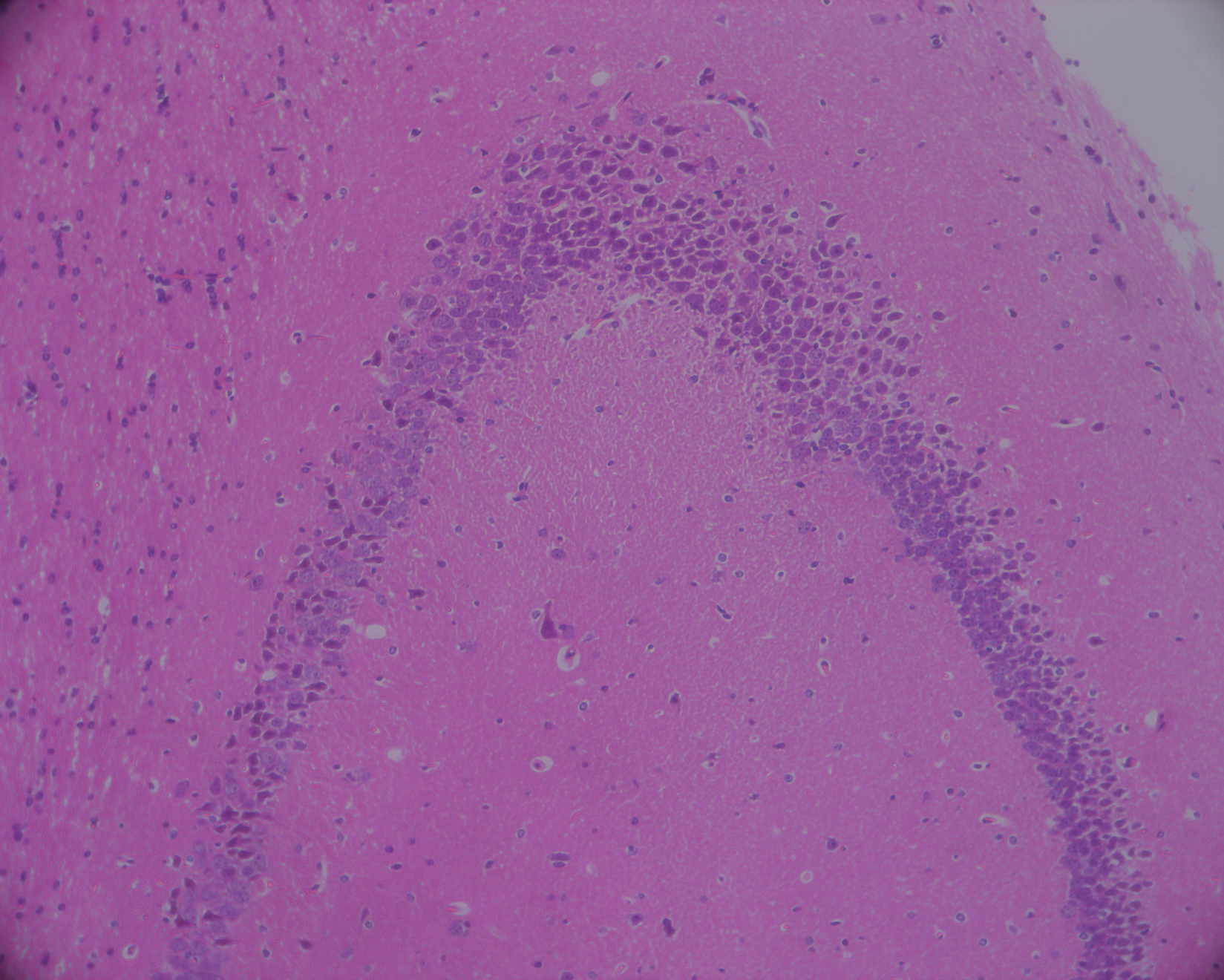

Supplement: S1 Data — (ZIP) [file pone.0311207.s001.zip › HE RSP+SSA.tif]

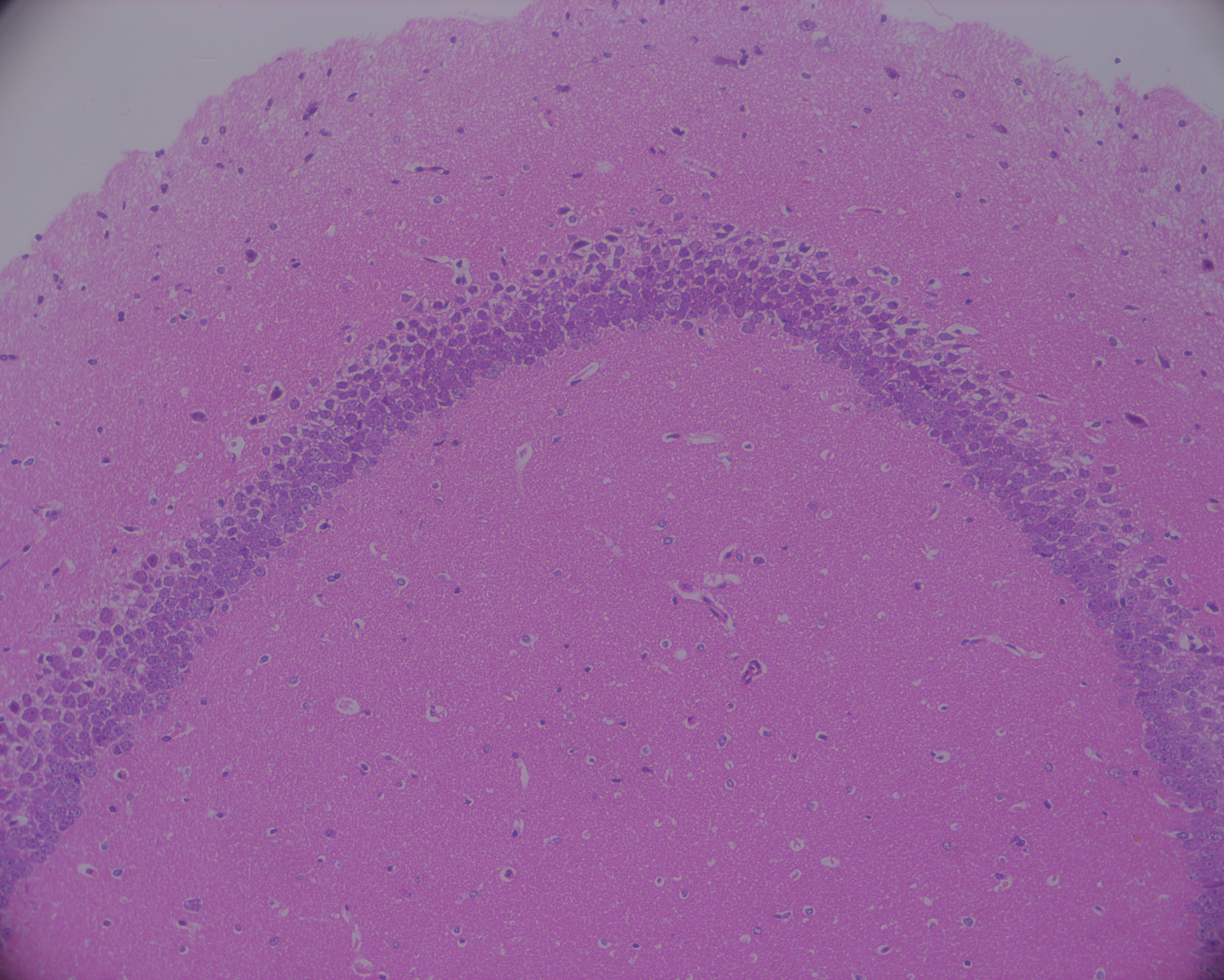

Supplement: S1 Data — (ZIP) [file pone.0311207.s001.zip › HE SSA.tif]

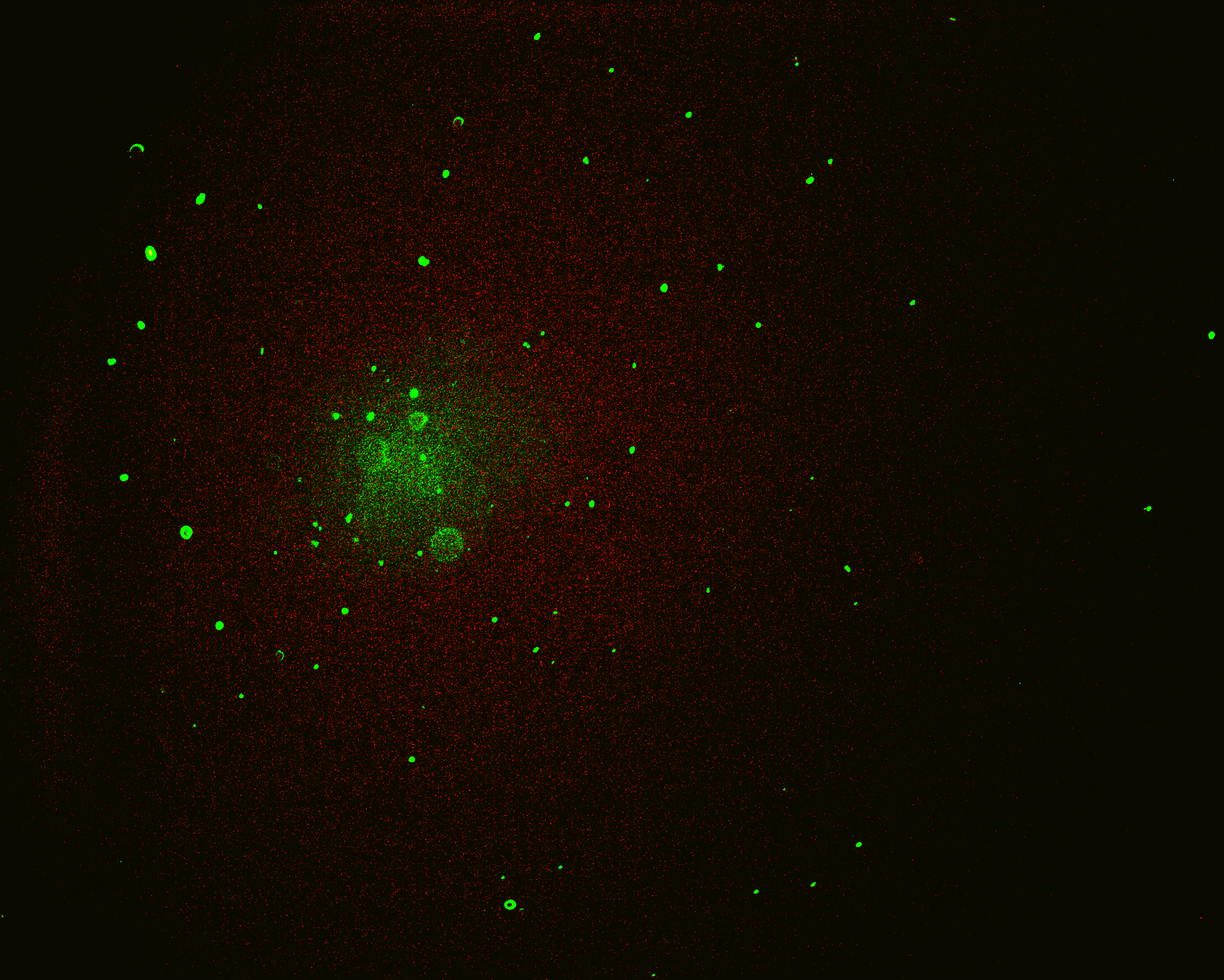

Supplement: S1 Data — (ZIP) [file pone.0311207.s001.zip › ROS CG .tif]

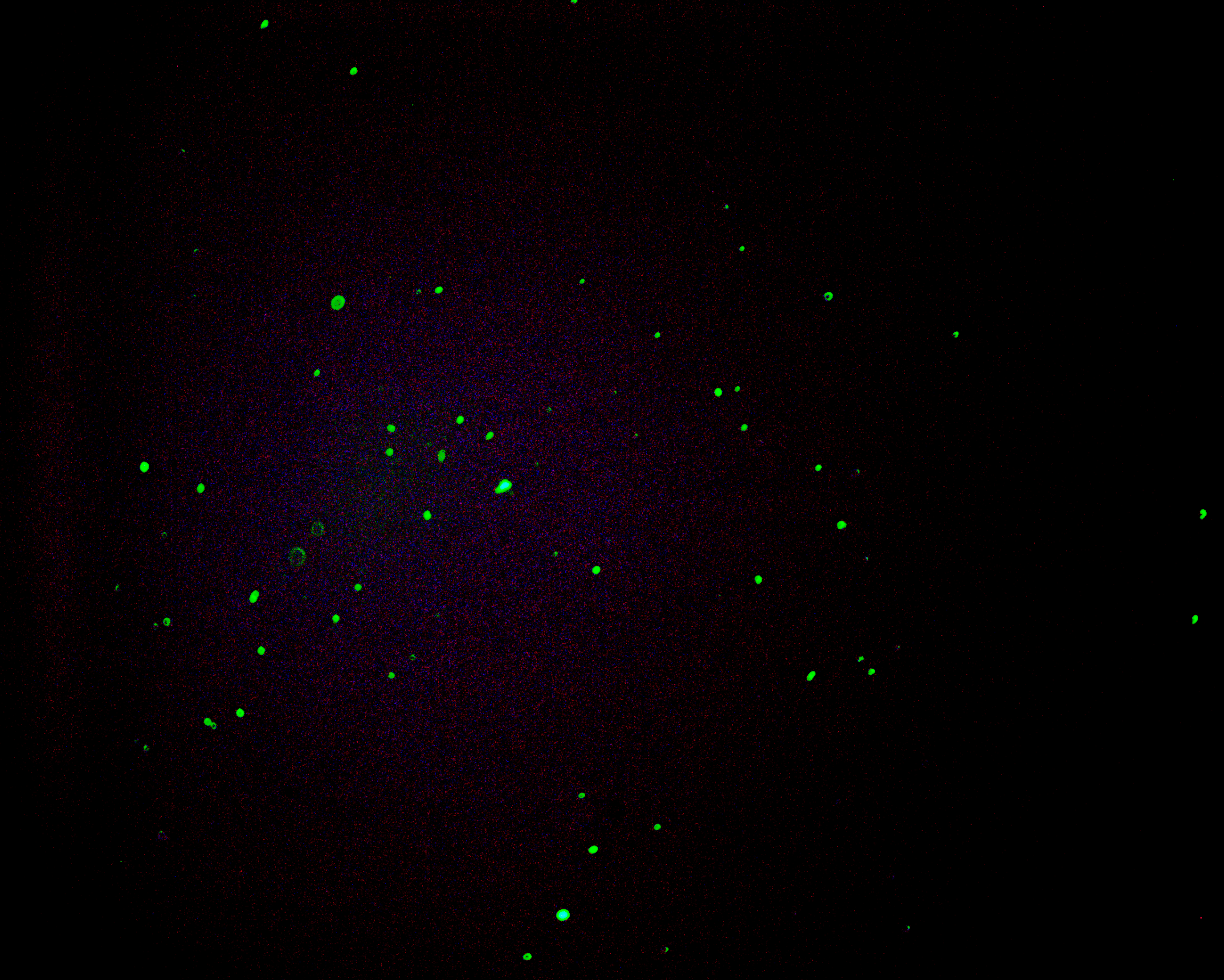

Supplement: S1 Data — (ZIP) [file pone.0311207.s001.zip › ROS RSP.tif]

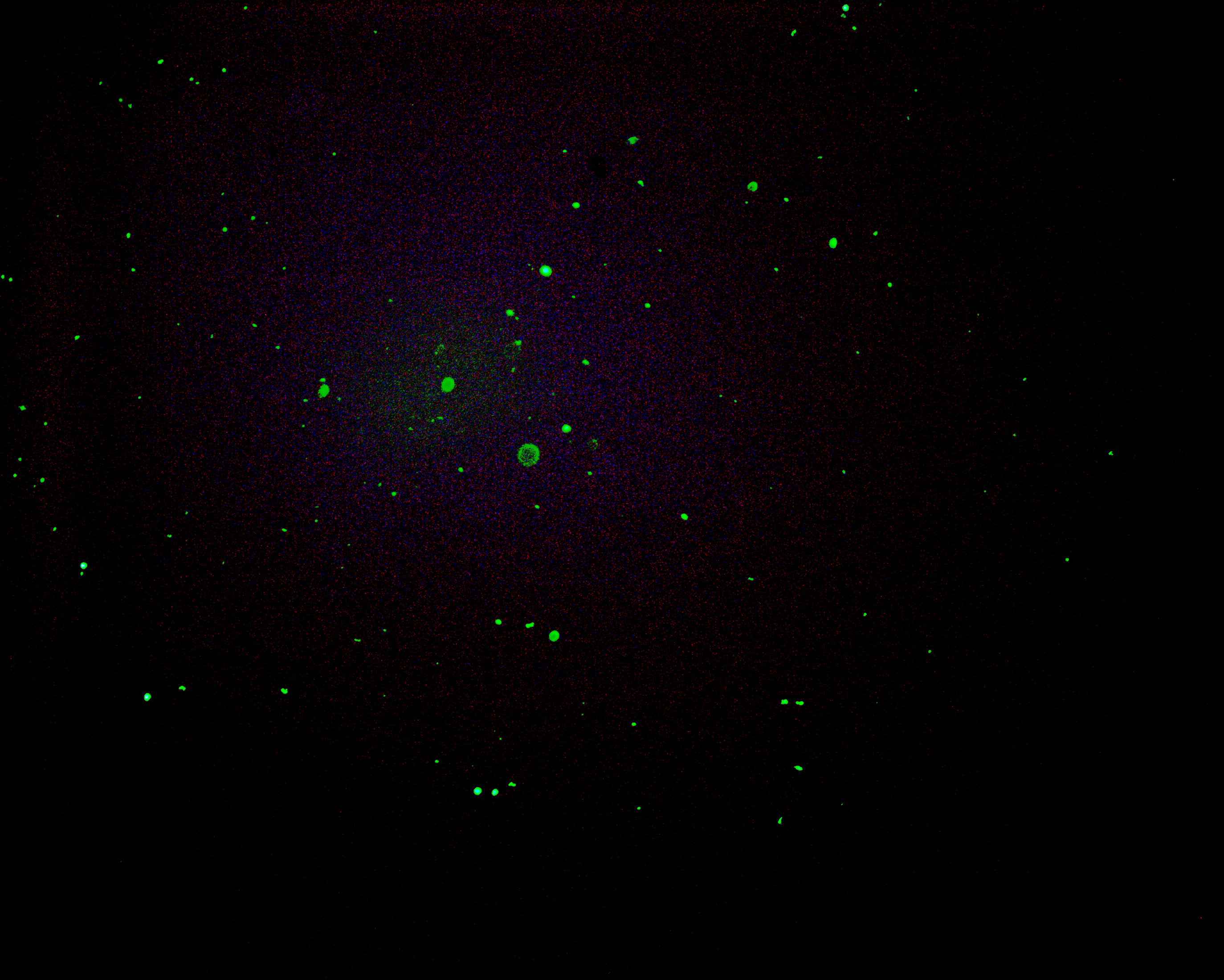

Supplement: S1 Data — (ZIP) [file pone.0311207.s001.zip › ROS RSP+SSA.tif]

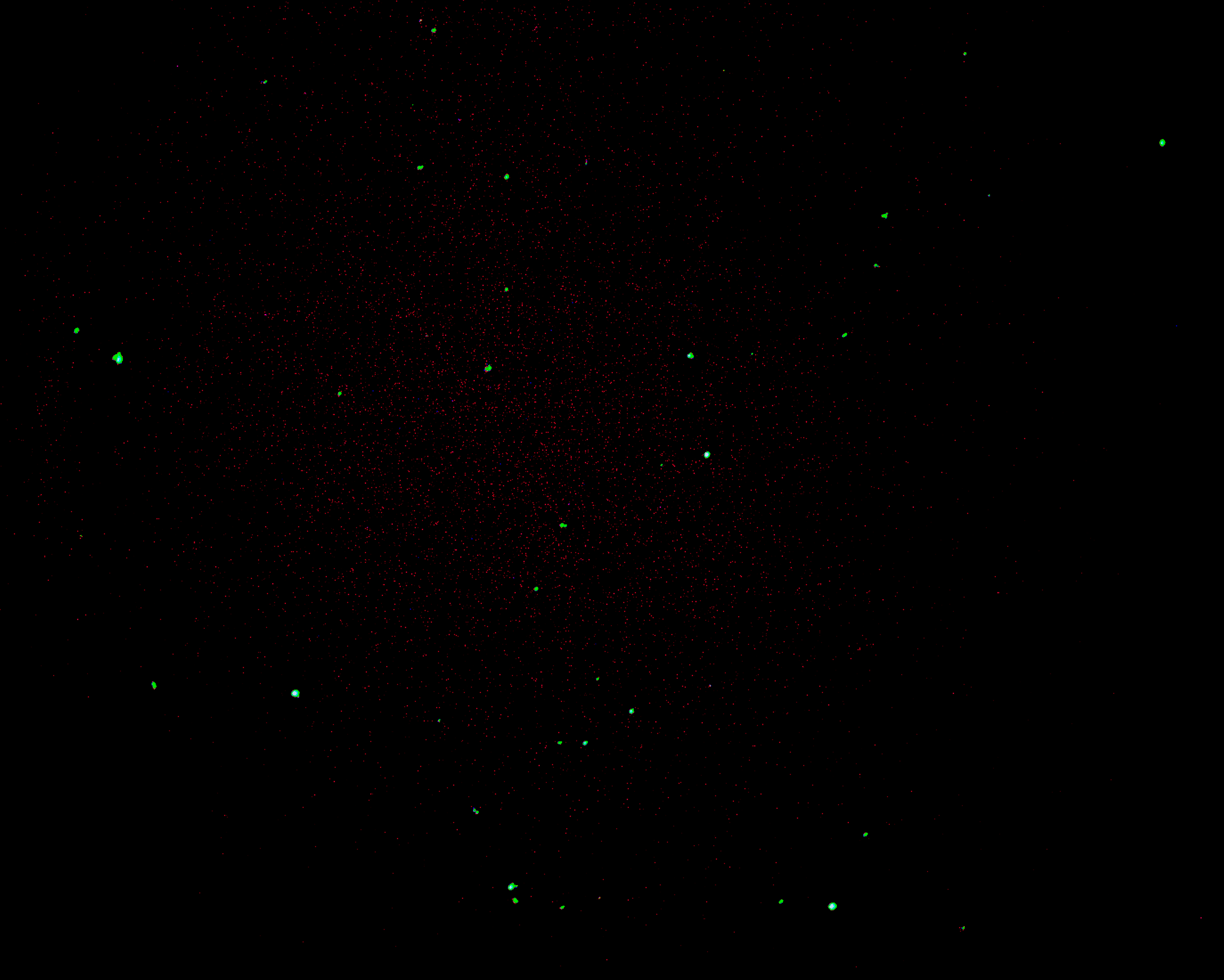

Supplement: S1 Data — (ZIP) [file pone.0311207.s001.zip › ROS SSA.tif]

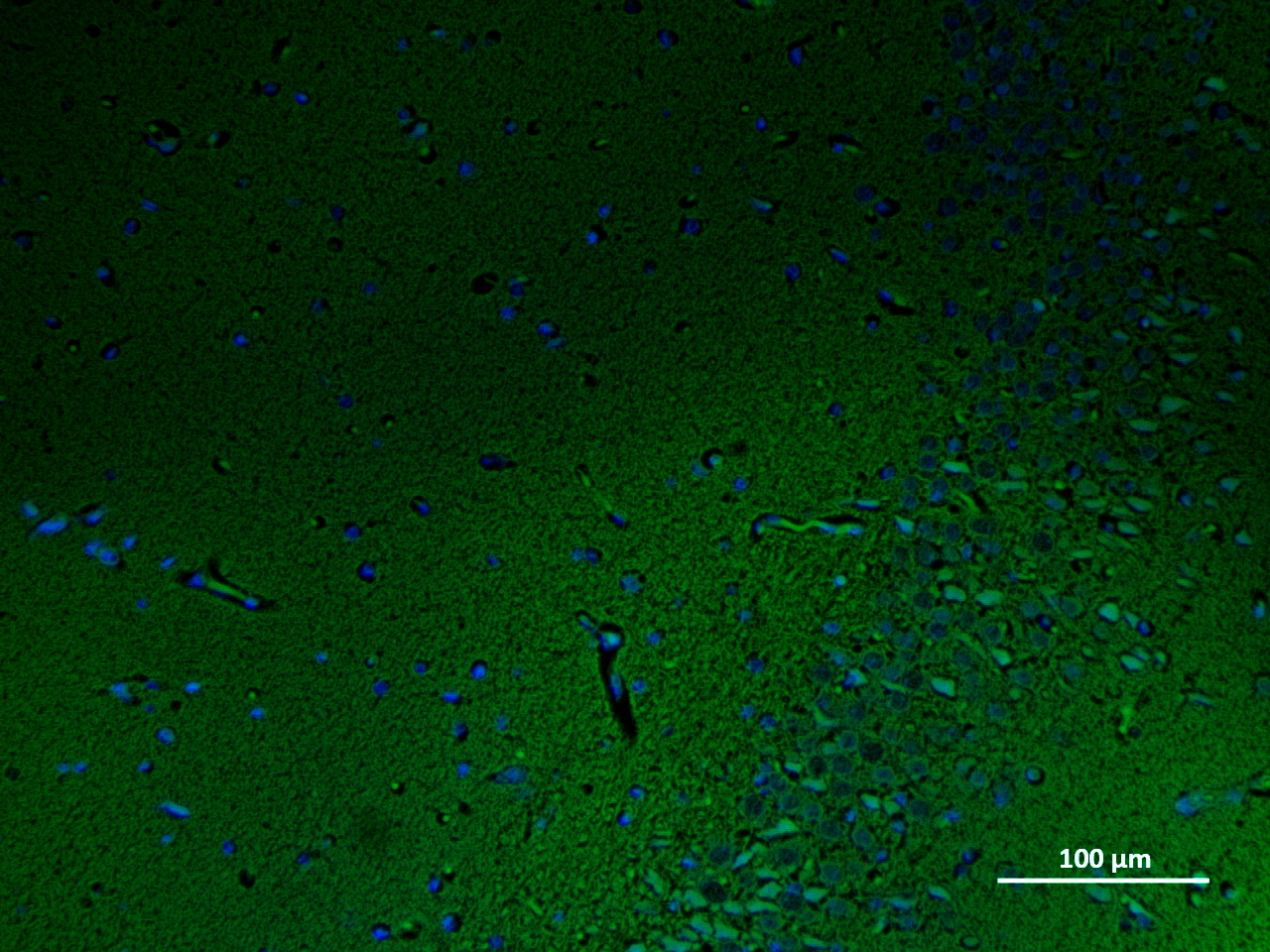

Supplement: S1 Data — (ZIP) [file pone.0311207.s001.zip › RSP FJB-1 200.jpg]

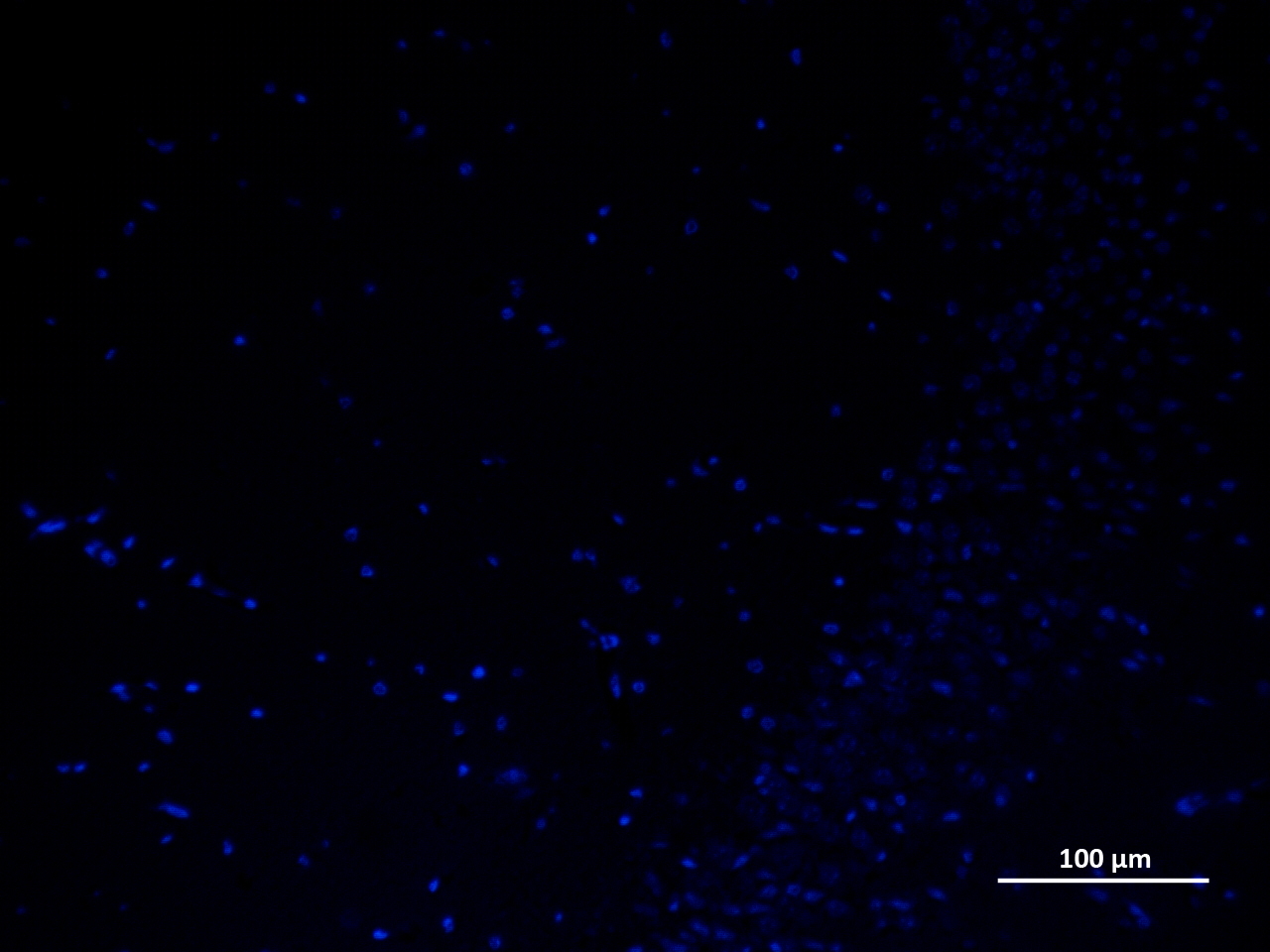

Supplement: S1 Data — (ZIP) [file pone.0311207.s001.zip › RSP FJB-1-1 200.jpg]

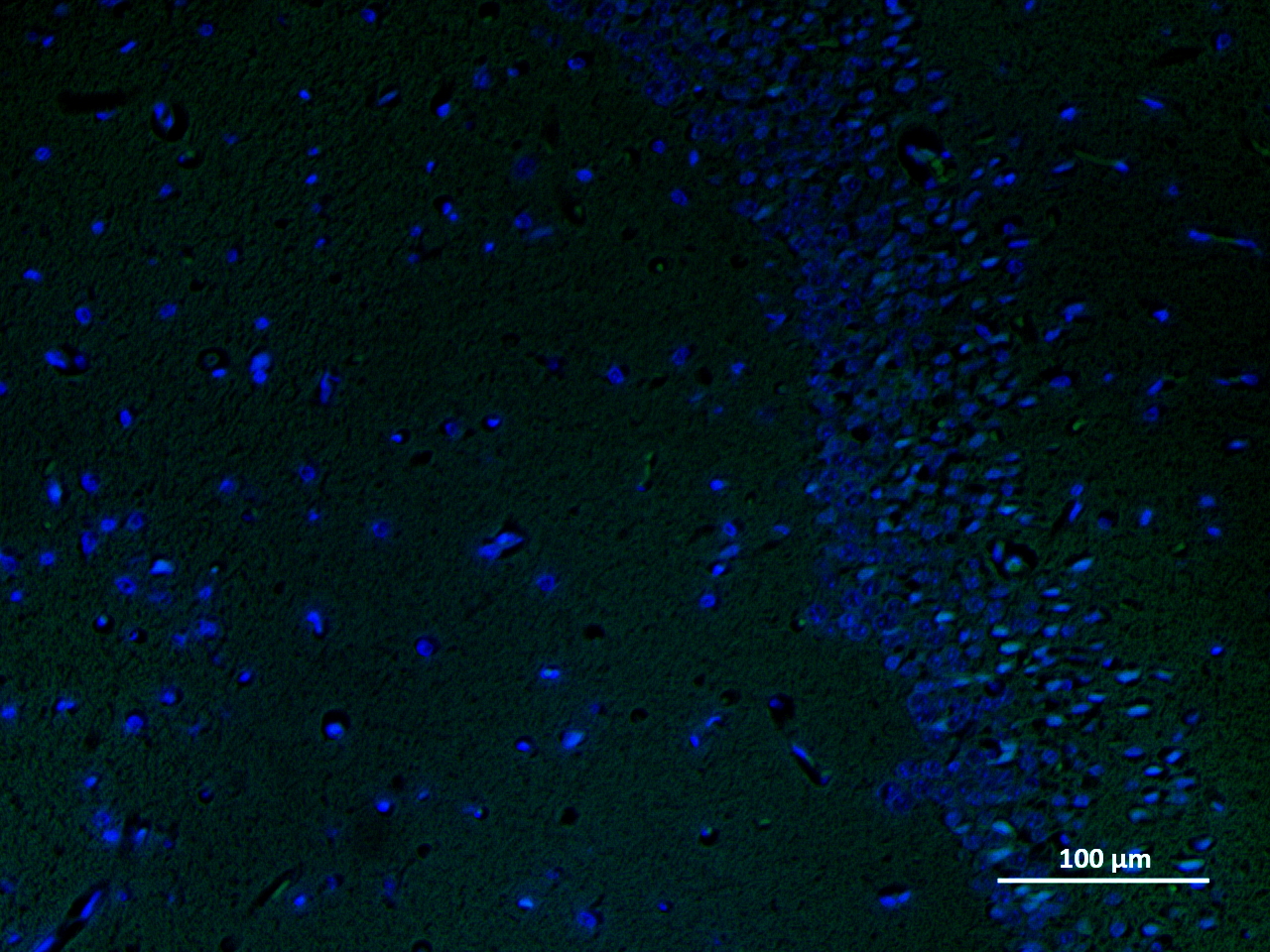

Supplement: S1 Data — (ZIP) [file pone.0311207.s001.zip › RSP+SSA FJB-1 200 .jpg]

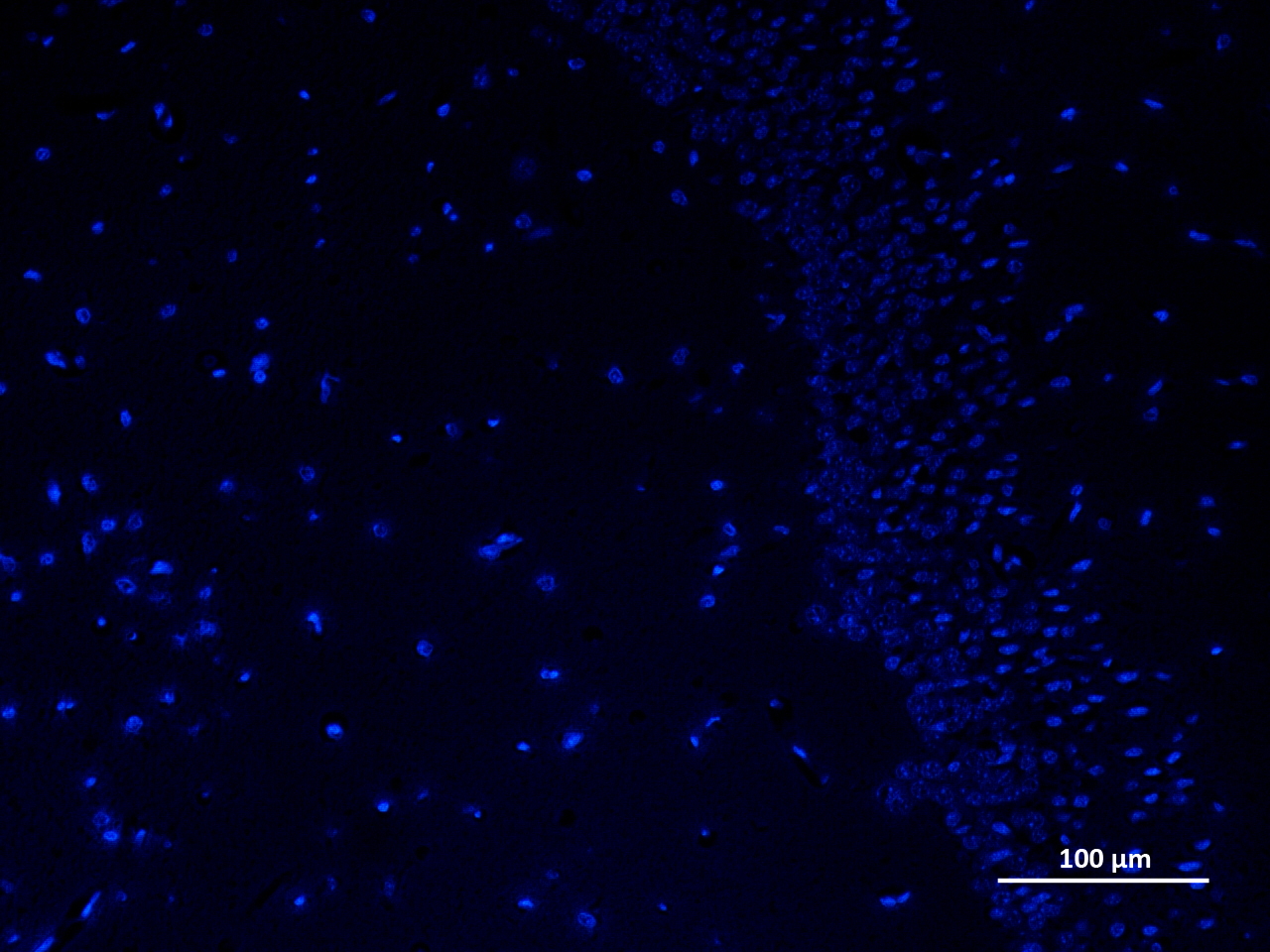

Supplement: S1 Data — (ZIP) [file pone.0311207.s001.zip › RSP+SSA FJB-1-1 200 .jpg]

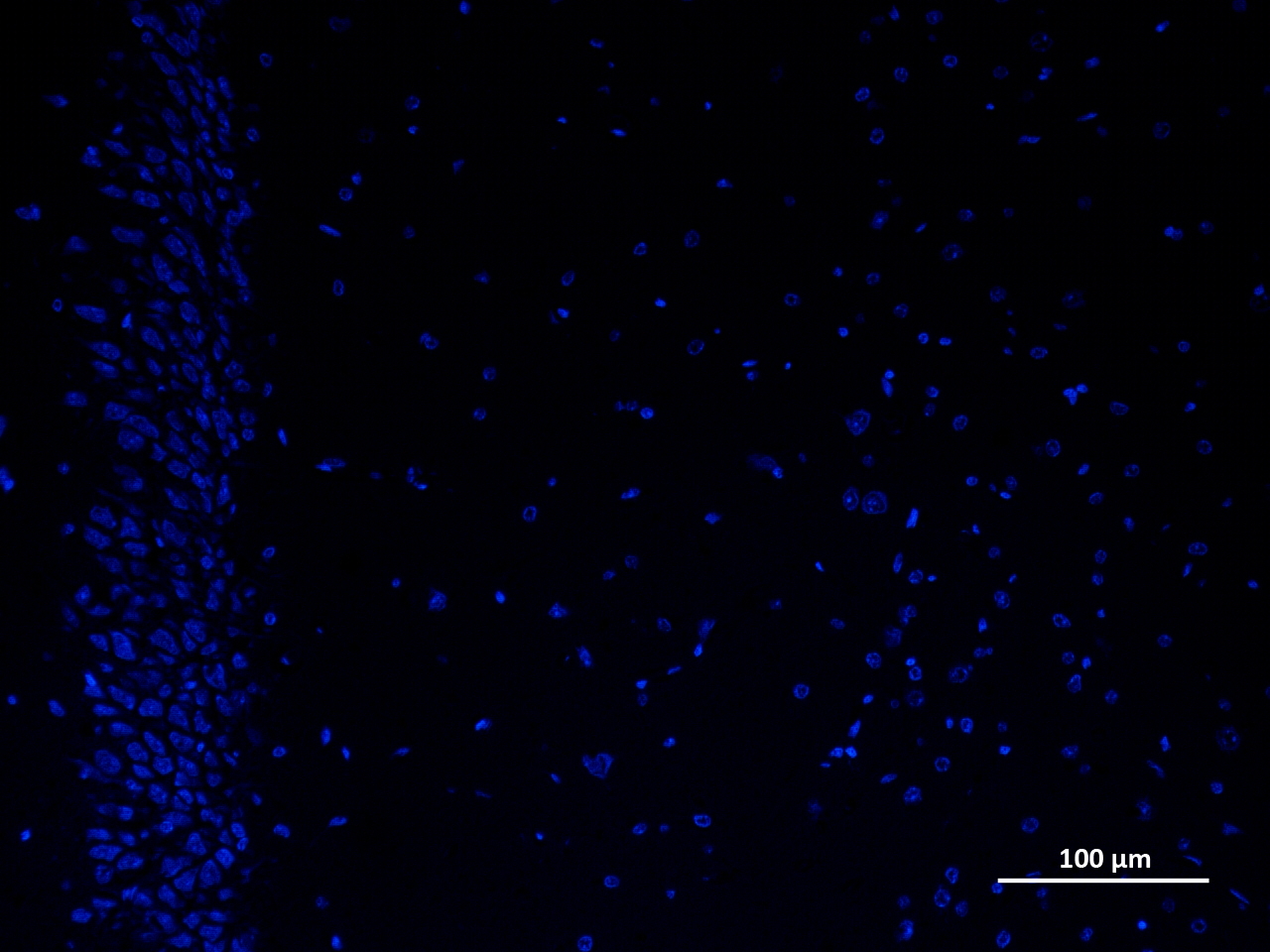

Supplement: S1 Data — (ZIP) [file pone.0311207.s001.zip › SSA FJB-1-1 200 .jpg]

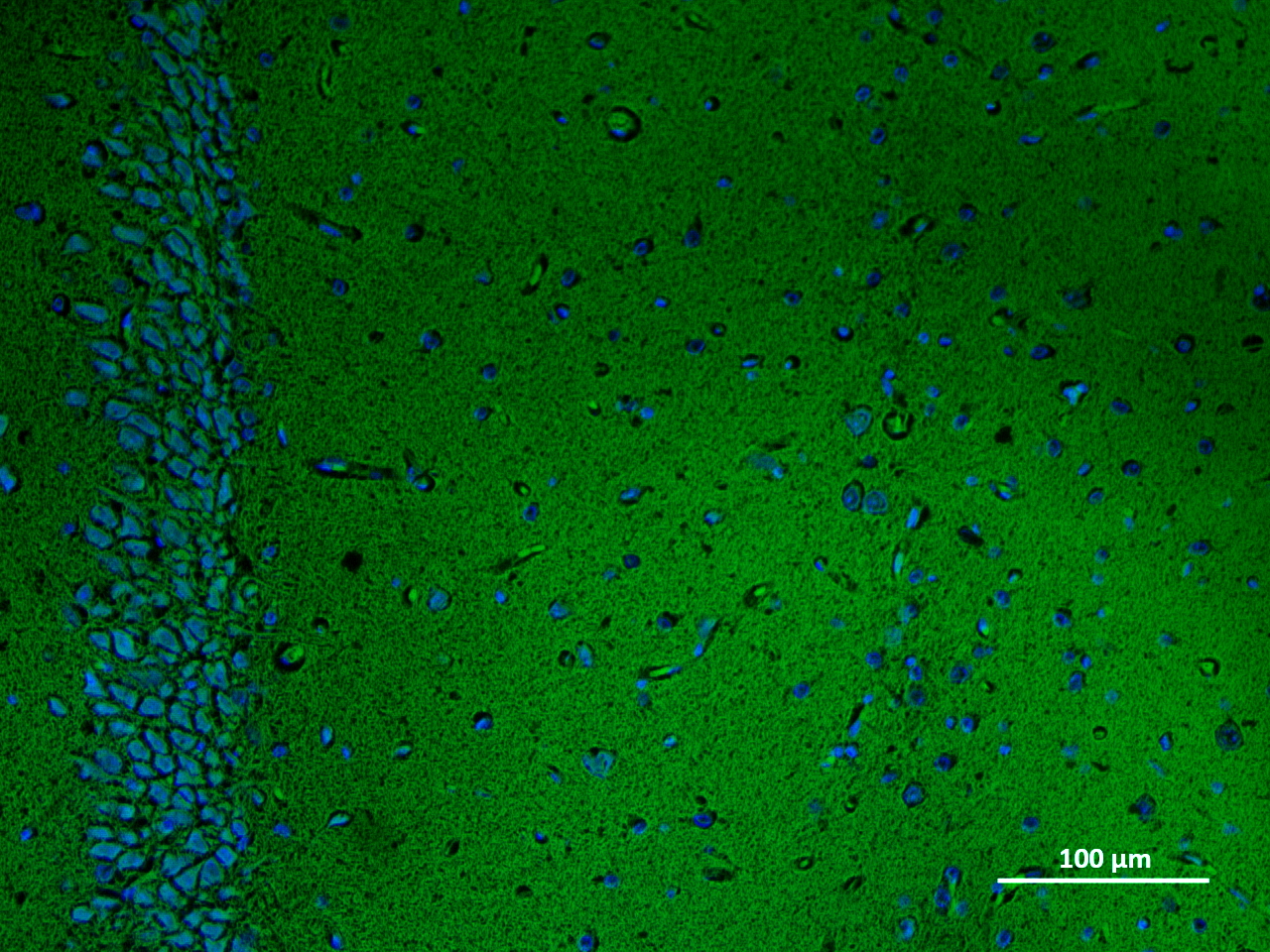

Supplement: S1 Data — (ZIP) [file pone.0311207.s001.zip › SSA FJB-1200 (1).jpg]

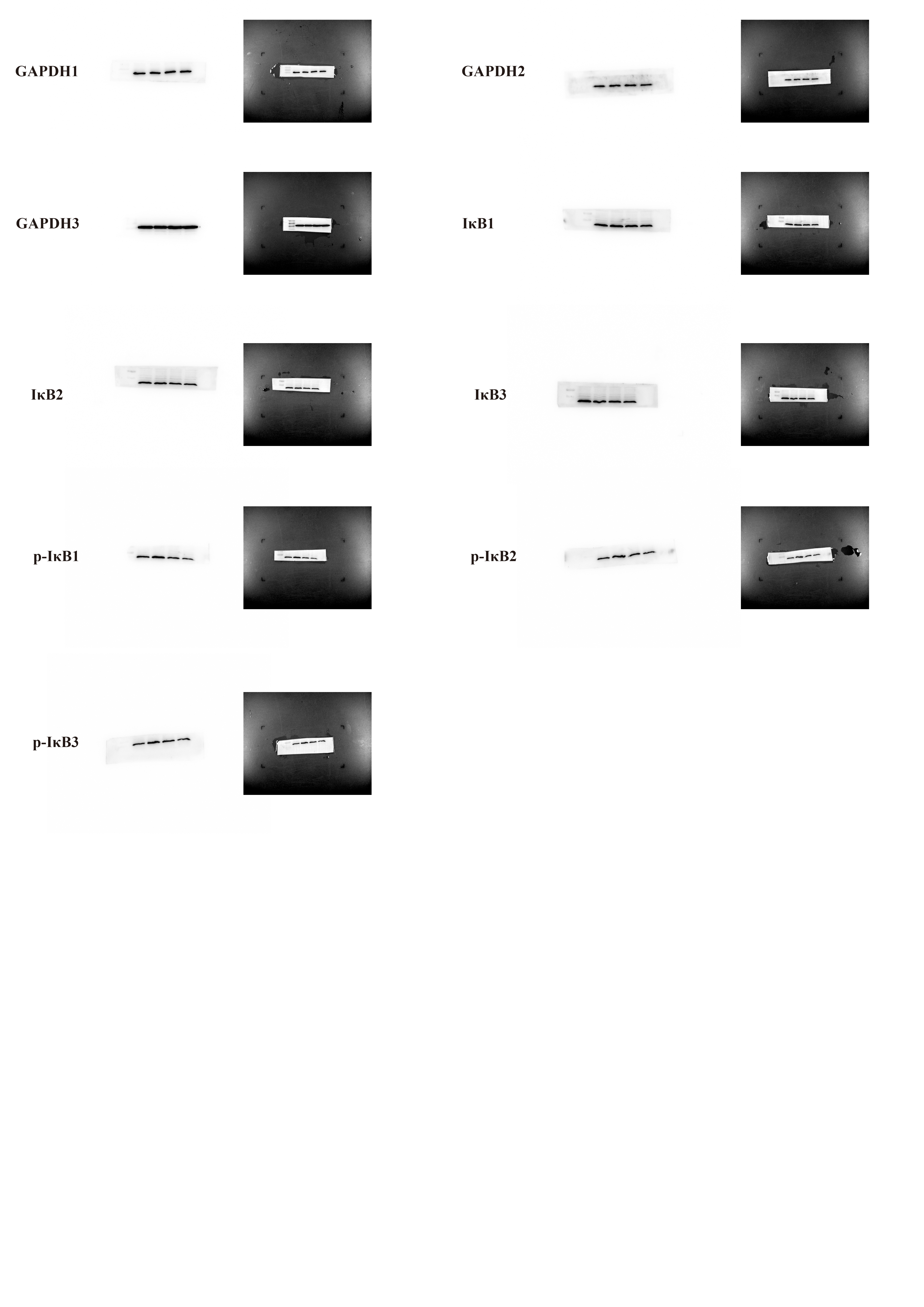

Supplement: S1 Data — (ZIP) [file pone.0311207.s001.zip › Supplement-figure6 C (WB).tif]
